# miRNA involvement in cell cycle regulation in colorectal cancer cases

**DOI:** 10.18632/genesandcancer.167

**Published:** 2018-01

**Authors:** Lila E. Mullany, Jennifer S. Herrick, Lori C. Sakoda, Wade Samowitz, John R. Stevens, Roger K. Wolff, Martha L. Slattery

**Affiliations:** ^1^ Division of Epidemiology, University of Utah, Salt Lake City, Ut, USA; ^2^ Division of Research, Kaiser Permanente Northern California, CA, USA; ^3^ Department of Pathology, University of Utah, Salt Lake City, Ut, USA; ^4^ Department of Mathematics and Statistics, Utah State University, Logan, Ut, USA

**Keywords:** cell cycle, colorectal, cancer, miRNA

## Abstract

Uncontrolled cell replication is a key component of carcinogenesis. MicroRNAs (miRNAs) regulate genes involved in checkpoints, DNA repair, and genes encoding for key proteins regulating the cell cycle. We investigated how miRNAs and mRNAs in colorectal cancer subjects interact to regulate the cell cycle.

Using RNA-Seq data from 217 individuals, we analyzed differential expression (carcinoma minus normal mucosa) of 123 genes within the cell cycle pathway with differential miRNA expression, adjusting for age and sex. Multiple comparison adjustments for gene/miRNA associations were made at the gene level using an FDR <0.05. Differentially expressed miRNAs and mRNAs were tested for associations with colorectal cancer survival. MRNA and miRNA sequences were compared to identify seed region matches to support biological interpretation of the observed associations.

Sixty-seven mRNAs were dysregulated with a fold change (FC) <0.67 or >1.50. Thirty-two mRNAs were associated with 48 miRNAs; 102 of 290 total associations had identified seed matches; of these, ten had negative beta coefficients. Hsa-miR-15a-5p and hsa-miR-20b-5p were associated with colorectal cancer survival with an FDR <0.05 (HR 0.86 95% CI 0.79, 0.94; HR 0.83 95% CI 0.75, 0.91 respectively).

Our findings suggest that miRNAs impact mRNA translation at multiple levels within the cell cycle.

## INTRODUCTION

The eukaryotic cell cycle can be divided in four major phases: G_1_, Synthesis (S), in which DNA is synthesized, G_2_, and Mitosis (M). Progression from one phase of the cell cycle to the next is controlled principally by a family of proteins called cyclins, which bind with and activate cyclin-dependent kinases (CDKs). Cyclins and CDKs form heterodimeric protein kinase complexes that are crucial for regulating specific steps in the cell cycle. Cyclins, which increase and decrease in concentration during different steps in the cell cycle, are the regulatory subunit of the complexes [1]. CDKs, which are serine/threonine kinases, are the catalytic subunits expressed in relatively stable amounts throughout the cell cycle [2]; it is their association with specific cyclins that determines whether the cell cycle progresses. CDKs have no catalytic activity of their own and so must be associated with a cyclin to phosphorylate different proteins.

Three main classes of cyclin-CDK complexes exist: G_1_, S, and mitotic complexes [1]. Each complex can phosphorylate specific groups of proteins, enabling coordinated gene expression at every step of the cell cycle. Cyclins D1, D2, and D3 bind to CDK4 and CDK6 to form the cyclin-CDK complexes necessary for G_1_ entry. These complexes phosphorylate the retinoblastoma protein (Rb), which blocks E2F transcription factors from activating gene expression [2]. This phosphorylation inactivates Rb, and allows E2F proteins to transcribe genes whose products are necessary for S-phase entry, namely cyclins A and E, and CDC25. Cyclin E binds to CDK2 to promote the transition from G_1_ to S phase by phosphorylating and inhibiting numerous proteins, including Rb and p21, which inhibits cyclin E as well as E2F [2, 3]. Cyclin E-CDK2 also phosphorylates components of the prereplication complex to initiate DNA replication [3]. Cyclin A2-CDK4 peaks during S phase and is thought to control DNA replication [3]. In late G_2_, cyclin A1 binds with CDK1 to promote M phase entry, which is primarily regulated by cyclin B-CDK1 complex [2]. CDK7, along with cyclin H, acts as a CDK activating kinase throughout the cell cycle [2].

Cyclin/cyclin-dependent kinase complexes and three supervisory restriction points, the G_1_/S, the G_2_/M and the metaphase checkpoints, are the predominant mechanisms of cell cycle regulation [4]. Uncontrolled growth is the hallmark of cancer, and as such perturbations in the cell cycle that downregulate cell cycle inhibitors, such as Rb, or upregulate cell cycle promoters, such as CDK activators, contribute to carcinogenesis [2]. MiRNAs, small, non-coding regulatory molecules, have been long established as post-transcriptional regulators of mRNA expression [5, 6]. MiRNAs have further been identified as a means of cell cycle control, through their involvement in the regulation of checkpoints as well as DNA repair [4, 7], and through the downregulation of cyclins, CDKs, cyclin-dependent kinase inhibitors (CKIs) and Rb [7]. In this way, miRNAs can act as oncogenes as well as tumor suppressors. Transcription Factors (TFs), including MYC and members of the E2F family, and miRNAs, such as the miR-17~92 and miR-106b~25 clusters, form feed-forward, feedback, and autoregulatory loops, further complicating the regulation of the cell cycle [4, 7, 8].

In this investigation, we identified differentially expressed cell cycle genes as well as miRNAs whose differential expression is associated with mRNA differential expression. Additionally, we tested whether these mRNAs and miRNAs are associated with altered colorectal cancer survival. We hypothesized that miRNAs are able to influence colorectal cancer outcomes through their involvement in regulating the expression of genes participating in the cell cycle pathway.

## RESULTS

Of the 217 participants, 169 were diagnosed with colon cancer and 48 were diagnosed with a rectal tumor (Table 1). Slightly more than half of the study population were male (54.4%) and on average, participants were aged 64.8 years at diagnosis. The largest proportion of study participants were non-Hispanic white (74.2%), with the remainder being non-Hispanic black (3.7%), Hispanic (6.5%), and of unknown race (15.7%). Twenty-nine participants had an MSI tumor; 92 (42.6%) study subjects were dead and 124 (57.4%) were alive at the end of follow-up, which was at least 5 years.

**Table 1:** Description of Study Population

		*N*	%
Site			
	Colon	169	77.9
	Rectal	48	22.1
Sex			
	Male	118	54.4
	Female	99	45.6
Age			
	Mean (SD)	64.8	10.1
Race			
	non-Hispanic White	161	74.2
	Hispanic	14	6.5
	non-Hispanic Black	8	3.7
	Unknown	34	15.7
AJCC Stage			
	1	58	27.1
	2	61	28.5
	3	72	33.6
	4	23	10.8
Tumor Phenotype			
	*TP53* mutated	103	47.5
	*KRAS* mutated	69	31.8
	*BRAF* mutated	21	10.1
	CIMP High	45	20.7
	MSI	29	13.4
Vital Status			
	Dead	92	42.6
	Alive	124	57.4

**Figure 1:**
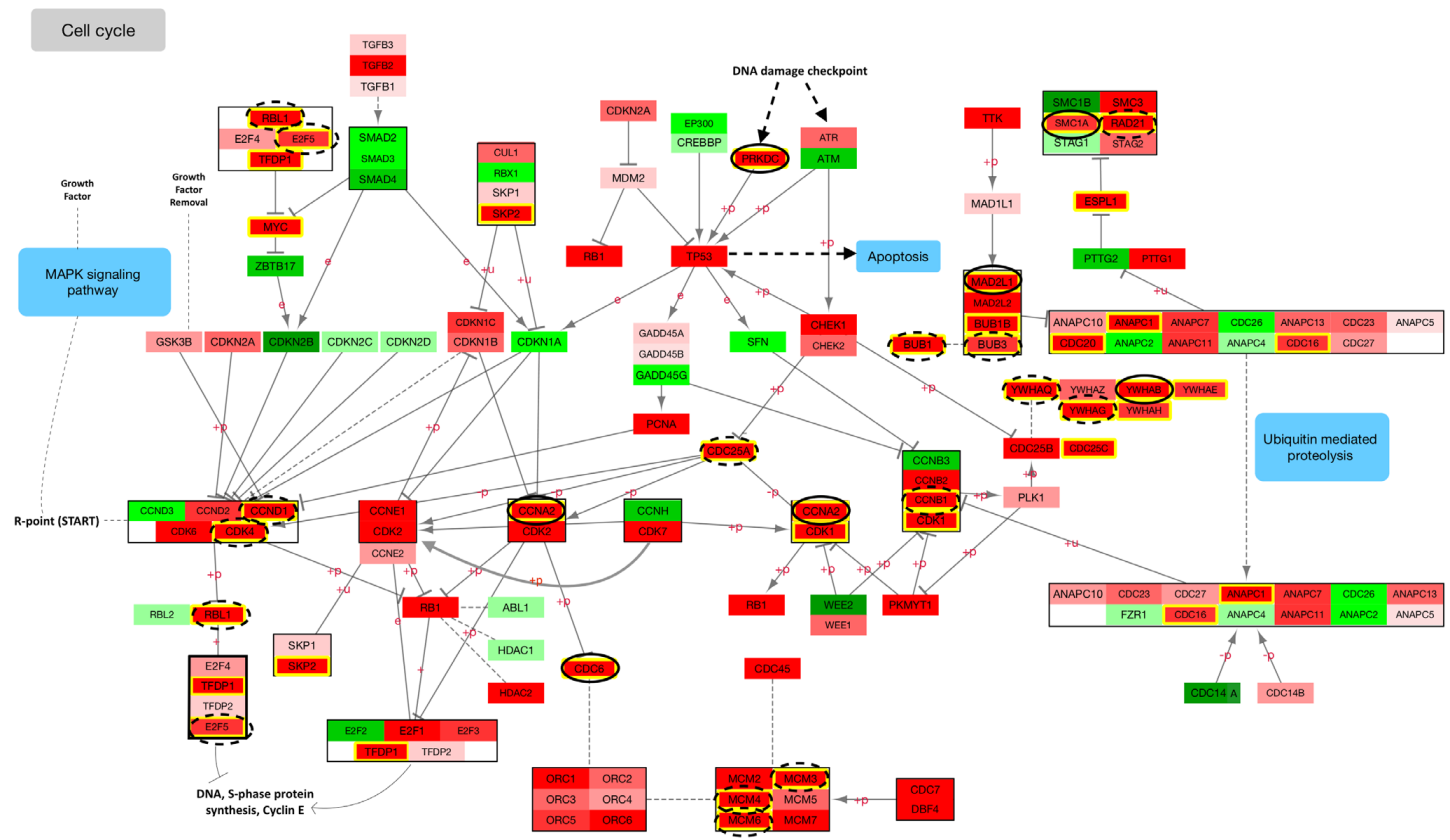
Downregulated mRNAs are shown in green, with the darkest green being < 0.67; upregulated mRNAs are shown in red, with the darkest red being > 1.50 MRNAs that were associated significantly with differential miRNA expression are highlighted in yellow. Those with identified seed matches are circled: mRNAs associated with negative beta coefficients have solid circles and those with positive beta coefficients have dashed circles.

We identified differentially expressed mRNAs for overall colorectal cancer, MSS-specific tumors, and MSI-specific tumors. For overall colorectal cancer, 110 of the 123 cell cycle mRNAs remained differentially expressed after adjustment for multiple comparisons (Figure 1). Of these 110 mRNAs, 54.5% (*n* = 67) were differentially expressed with a FC > 1.50 or < 0.67 (Table 2) with all but four being up-regulated. Among MSS tumors only, one mRNA (*ANAPC13*) was significantly differentially expressed that was not associated with overall colorectal cancer, and three mRNAs (*MCM5*, *MDM2*, and *WEE1*) were unique to MSI tumors when considering only genes with a FC > 1.50 or < 0.67 and correcting for multiple comparisons.

**Table 2:** Differentially expressed mRNAs with a fold change (FC) >1.50 or <0.67 and adjusted *p*-value <0.05

	Mean Expression				
Gene	Carcinoma	Normal Mucosa	Fold Change	*P*-Value	Adjusted *P*-Value
*ANAPC1*^1^	75.40	43.01	1.75	<0.001	<0.001
*ANAPC11*	11.96	7.71	1.55	<0.001	<0.001
*ANAPC13*^2^	26.31	17.35	1.52	<0.001	<0.001
*ANAPC7*^1^	65.84	43.85	1.50	<0.001	<0.001
*BUB1*	53.35	18.38	2.90	<0.001	<0.001
*BUB1B*	41.59	17.15	2.42	<0.001	<0.001
*BUB3*^1^	94.35	60.85	1.55	<0.001	<0.001
*CCNA2*	40.09	14.62	2.74	<0.001	<0.001
*CCNB1*	32.40	9.25	3.50	<0.001	<0.001
*CCNB2*	21.30	12.39	1.72	<0.001	<0.001
*CCND1*	317.79	122.64	2.59	<0.001	<0.001
*CCND2*	773.45	483.06	1.60	<0.001	<0.001
*CCNE1*	8.70	4.80	1.81	<0.001	<0.001
*CDC14A*	16.20	31.59	0.51	<0.001	<0.001
*CDC16*^1^	90.37	58.88	1.53	<0.001	<0.001
*CDC20*	22.28	10.17	2.19	<0.001	<0.001
*CDC25A*	21.63	10.61	2.04	<0.001	<0.001
*CDC25B*	169.88	60.96	2.79	<0.001	<0.001
*CDC25C*	7.95	3.46	2.30	<0.001	<0.001
*CDC45*	14.50	6.61	2.19	<0.001	<0.001
*CDC6*	32.30	11.33	2.85	<0.001	<0.001
*CDC7*	19.56	10.85	1.80	<0.001	<0.001
*CDK1*	41.36	11.94	3.46	<0.001	<0.001
*CDK2*	45.70	24.96	1.83	<0.001	<0.001
*CDK4*	66.65	26.90	2.48	<0.001	<0.001
*CDK6*	289.15	166.12	1.74	<0.001	<0.001
*CDK7*	23.94	13.17	1.82	<0.001	<0.001
*CDKN1C*^1^	5.45	3.38	1.61	<0.001	<0.001
*CDKN2B*	24.21	74.65	0.32	<0.001	<0.001
*CHEK1*	37.56	15.63	2.40	<0.001	<0.001
*DBF4*	23.05	11.73	1.97	<0.001	<0.001
*E2F1*	27.36	9.14	2.99	<0.001	<0.001
*E2F3*^1^	57.78	34.90	1.66	<0.001	<0.001
*E2F5*^1^	46.80	30.87	1.52	<0.001	<0.001
*ESPL1*	39.52	18.82	2.10	<0.001	<0.001
*HDAC2*	102.64	58.70	1.75	<0.001	<0.001
*MAD2L1*	15.96	4.92	3.24	<0.001	<0.001
*MAD2L2*	9.52	5.51	1.73	<0.001	<0.001
*MCM2*	44.16	17.25	2.56	<0.001	<0.001
*MCM3*	107.53	41.43	2.60	<0.001	<0.001
*MCM4*	115.85	43.65	2.65	<0.001	<0.001
*MCM5*^3^	78.55	50.31	1.56	<0.001	0.002
*MCM6*	55.02	23.15	2.38	<0.001	<0.001
*MCM7*	135.12	64.18	2.11	<0.001	<0.001
*MDM2*^3^	358.47	229.39	1.56	<0.001	<0.001
*MYC*	181.11	49.00	3.70	<0.001	<0.001
*ORC1*	13.40	7.68	1.74	<0.001	<0.001
*ORC5*^1^	23.07	15.12	1.53	<0.001	<0.001
*ORC6*	13.15	4.62	2.84	<0.001	<0.001
*PCNA*	37.26	12.83	2.91	<0.001	<0.001
*PKMYT1*	45.18	16.72	2.70	<0.001	<0.001
*PRKDC*	927.46	395.35	2.35	<0.001	<0.001
*PTTG1*	14.78	5.48	2.70	<0.001	<0.001
*RAD21*	257.68	139.20	1.85	<0.001	<0.001
*RB1*^1^	104.85	58.44	1.79	<0.001	<0.001
*RBL1*	53.52	23.32	2.30	<0.001	<0.001
*SKP2*	49.31	24.13	2.04	<0.001	<0.001
*SMC1A*	208.24	133.73	1.56	<0.001	<0.001
*SMC1B*^1^	3.71	6.20	0.60	<0.001	<0.001
*SMC3*	123.45	66.72	1.85	<0.001	<0.001
*TFDP1*	119.20	54.04	2.21	<0.001	<0.001
*TGFB2*^1^	9.23	4.52	2.04	<0.001	<0.001
*TP53*	105.07	59.63	1.76	<0.001	<0.001
*TTK*	28.80	10.97	2.62	<0.001	<0.001
*WEE1*^3^	129.22	85.54	1.51	<0.001	<0.001
*WEE2*	1.33	2.11	0.63	0.002	0.002
*YWHAB*^1^	389.26	223.04	1.75	<0.001	<0.001
*YWHAE*^1^	214.66	141.14	1.52	<0.001	<0.001
*YWHAG*	241.75	119.30	2.03	<0.001	<0.001
*YWHAH*	102.59	66.00	1.55	<0.001	<0.001
*YWHAQ*	130.47	72.74	1.79	<0.001	<0.001

^1^Uniquely dysregulated in overall CRC and not in MSS or MSI.

^2^Uniquely dysregulated in MSS tumors.

^3^Uniquely dysregulated in MSI tumors.

**Figure 2:**
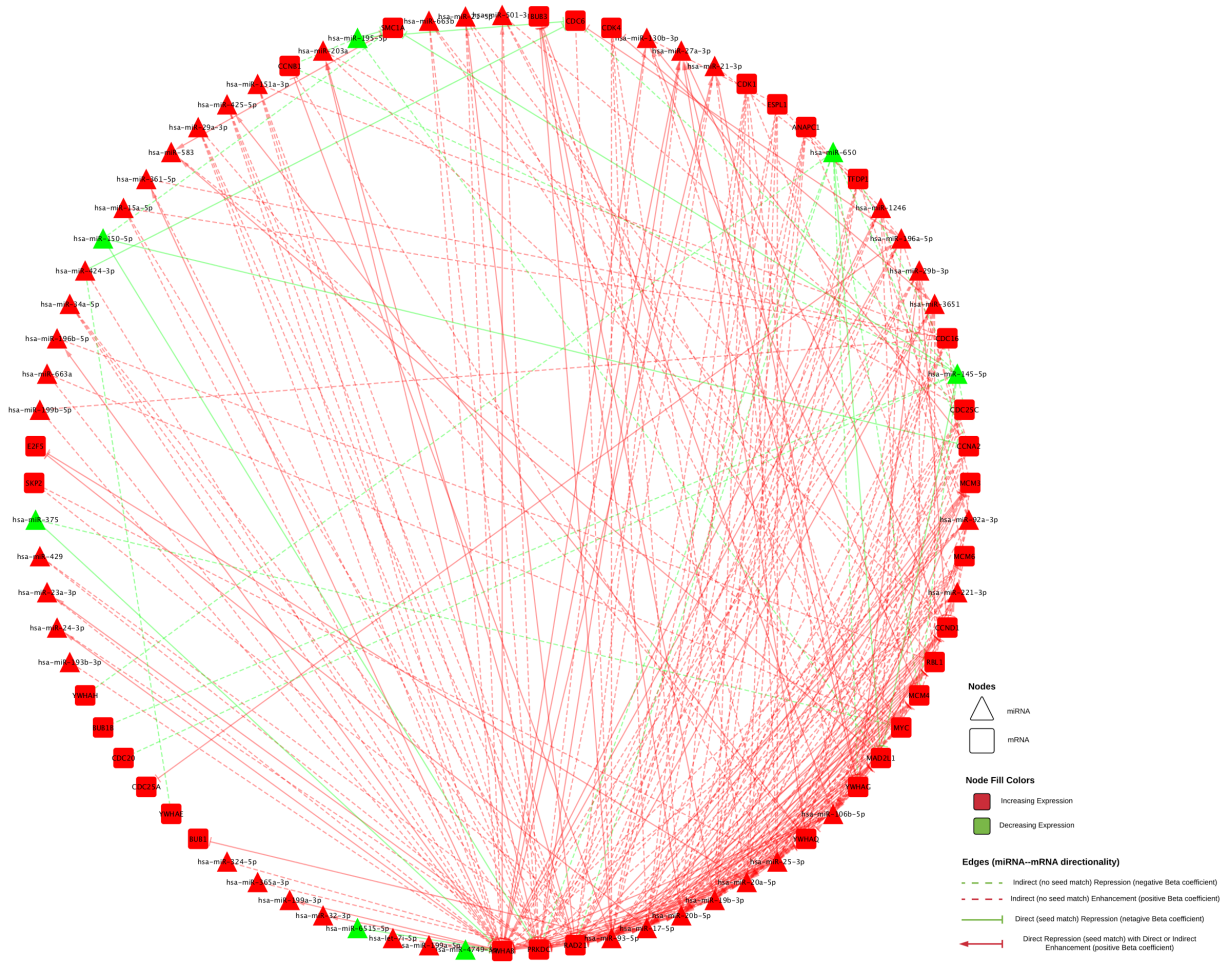
All miRNA-mRNA associations are shown MiRNAs are shown in triangles, mRNAs are shown in squares. Downregulated genes are shown in green and upregulated are shown in red. Positive beta coefficients are shown in red lines, negative beta coefficients are shown in green. Direct associations, those with identified seed matches are shown with a solid line and a stop (--|); those with a positive beta coefficient in addition to a seed match have an arrow (♢) leading from the mRNA to the miRNA.

When examining associations of differentially expressed mRNAs with differentially expressed miRNAs, we identified 290 unique interactions, comprising 32 mRNAs and 48 miRNAs with a FC < 0.67 or > 1.50 and an FDR of < 0.05 (Figure 2). All 32 dysregulated mRNAs were upregulated in colorectal cancer tissue compared to normal colorectal mucosa; 41 of the dysregulated miRNAs were upregulated and seven were downregulated in colorectal cancer tissue compared to normal colorectal mucosa. The majority of the 290 miRNA-mRNA associations (261, 90%) had positive beta coefficients; the remaining 29 (10%) interactions had negative beta coefficients. Additionally, we analyzed miRNA and mRNA 3’ UTR FASTA sequences for seed region matches. Ten of the 29 interactions with negative beta coefficients and 92 of the 261 interactions with positive beta coefficients had seed region matches between the miRNA and mRNA.

Increased differential expression of four mRNAs was associated with improved colorectal cancer survival prior to adjustment for multiple comparisons (Table 3); these findings did not remain significant after adjustment for multiple comparisons. Twelve miRNAs were associated with altered colorectal cancer survival. Two miRNAs, hsa-miR-145-5p (HR 1.13, 95% CI 1.10, 1.26) and hsa-miR-193b-3p (HR 1.10, 95% CI 1.01, 1.20), reduced colorectal cancer survival when expression of these miRNAs in carcinoma tissue was increased. Increased differential expression of ten miRNAs also was associated with improved colorectal cancer survival (Table 3). Two miRNAs, hsa-miR-15a-5p and hsa-miR-34a-5p, remained statistically significant after adjustment for multiple comparisons.

**Table 3:** MRNAs and miRNAs associated significantly with altered CRC survival^1^

mRNA	Q1	Q3	HR	(95% CI)	*P*-value	Q-value	FDR *P*-value
*CDC16*	3	58.87	0.69	(0.51, 0.93)	0.025	0.251	0.513
*CDC25A*	1.11	21.34	0.71	(0.54, 0.94)	0.029	0.251	0.513
*CHEK1*	7.2	34.54	0.56	(0.38, 0.83)	0.004	0.245	0.142
*E2F5*	0.81	30.08	0.59	(0.43, 0.81)	0.002	0.245	0.142

miRNA	Q1	Q3	HR	(95% CI)	*P*-value	Q-value	FDR *P*-value
hsa-miR-145-5p	-1.94	-0.15	1.13	(1.01, 1.26)	0.03	0.117	0.162
hsa-miR-15a-5p	-0.31	1.55	0.86	(0.79, 0.94)	0.001	0.117	0.022
hsa-miR-17-5p	0.95	2.4	0.91	(0.84, 0.98)	0.016	0.117	0.155
hsa-miR-193b-3p	-0.16	1.45	1.1	(1.01, 1.20)	0.035	0.117	0.166
hsa-miR-19b-3p	0.61	2.35	0.91	(0.84, 0.99)	0.024	0.117	0.162
hsa-miR-20a-5p	1.02	2.61	0.91	(0.84, 0.98)	0.019	0.117	0.155
hsa-miR-20b-5p	0.96	3.11	0.83	(0.75, 0.91)	0.000	0.117	0.009
hsa-miR-29a-3p	0.39	1.74	0.91	(0.84, 0.98)	0.015	0.117	0.155
hsa-miR-34a-5p	0.27	1.57	0.9	(0.84, 0.97)	0.005	0.117	0.082
hsa-miR-425-5p	-0.1	1.48	0.92	(0.85, 0.99)	0.043	0.117	0.172
hsa-miR-92a-3p	0.63	1.87	0.91	(0.83, 0.99)	0.029	0.117	0.162
hsa-miR-93-5p	0.71	1.99	0.93	(0.86, 0.99)	0.042	0.117	0.172

^1^MRNAs that were differentially expressed were tested for associations with survival. MiRNAs that were differentially expressed and associated with mRNA differential expression were tested for associations with survival.

**Figure 3:**
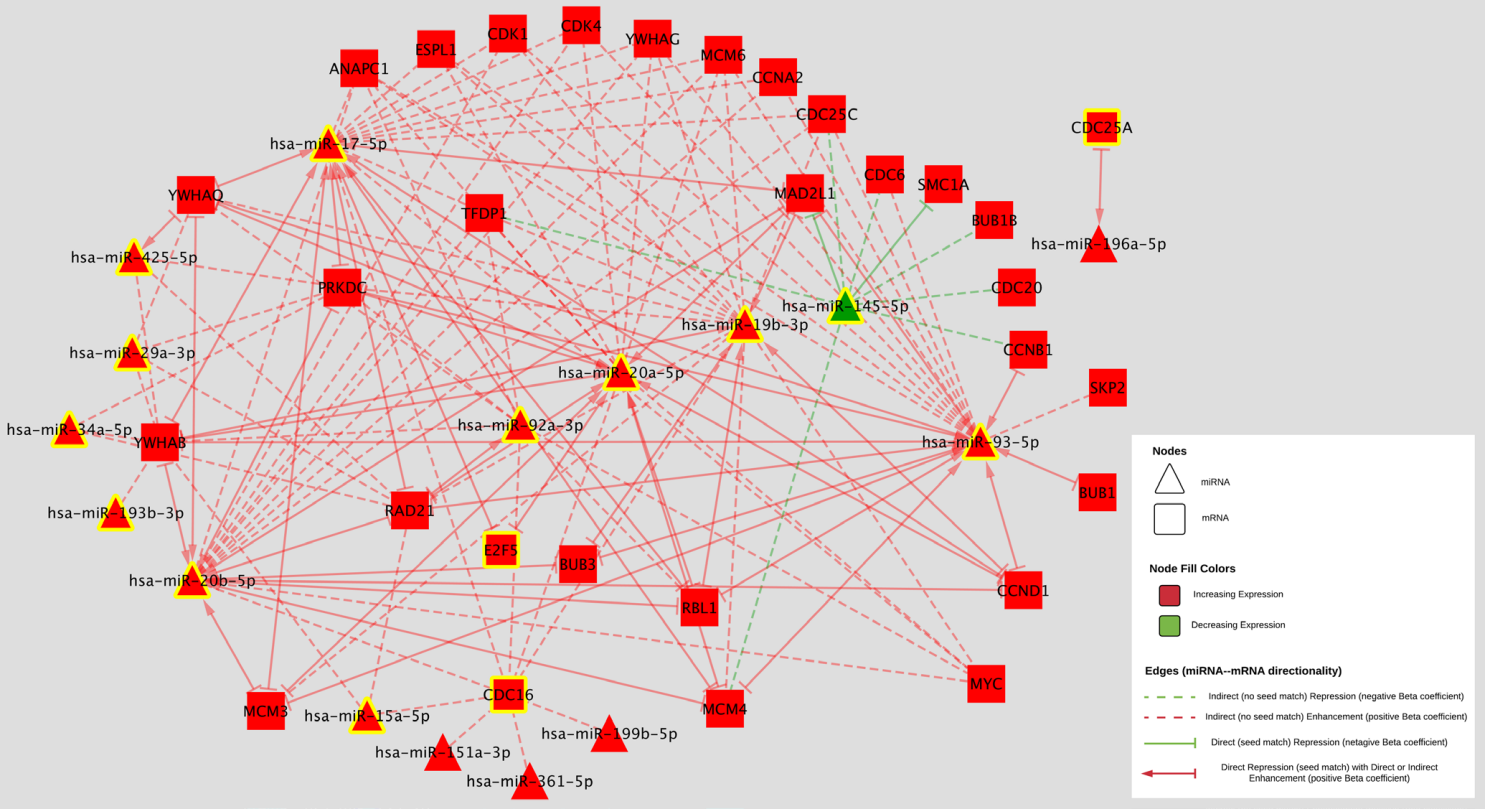
MiRNA-mRNA associations for those involving a miRNA or mRNA that was associated with an altered risk of colorectal cancer survival prior to adjustment for multiple comparisons are shown MiRNAs are shown in triangles, mRNAs are shown in squares. Downregulated genes are shown in green and upregulated are shown in red. Positive beta coefficients are shown in red lines, negative beta coefficients are shown in green. Direct associations, those with identified seed matches are shown with a solid line and a stop (--|); those with a positive beta coefficient in addition to a seed match have an arrow (♢) leading from the mRNA to the miRNA. Genes that were associated with altered risk of colorectal cancer survival are highlighted in yellow.

Three of the four mRNAs associated with altered survival prior to adjustment for multiple comparisons were associated with differential miRNA expression (Figure 3). Eleven of the twelve miRNAs associated with altered colorectal cancer survival prior to adjustment for multiple comparisons were upregulated in carcinoma tissue compared to normal colorectal mucosa and one was downregulated. Two mRNAs (*E2F5* and *CDC16*) that were associated with altered colorectal cancer survival were associated with six of the miRNAs associated with altered colorectal cancer survival: hsa-miR-15a-5p, hsa-miR-17-5p, hsa-miR-19b-3p, hsa-miR-20a-5p, hsa-miR-20b-5p, and hsa-miR-92a-3p. All of these miRNAs were associated with *CDC16* with a positive beta coefficient and had no identified seed matches; hsa-miR-17-5p and hsa-miR-20a-5p were associated with *E2F5* with a negative beta coefficient and had identified seed matches.

## DISCUSSION

Of the 124 cell cycle genes in the KEGG repository, 110 were statistically significantly differentially expressed for overall colorectal cancer after adjustment for multiple comparisons. Nineteen of these genes were downregulated in carcinoma tissue compared to normal colorectal mucosa, four of these with a FC < 0.67. The remaining 91 genes were upregulated, 63 of which had a FC > 1.50. Collectively, these genes regulate every point of the cell cycle, as evident in Figure 1. Additionally, mRNAs involved in every phase of the cell cycle were associated with differentially expressed miRNAs, many with identified seed matches, indicating their potential direct regulation by miRNAs.

Thirty-two mRNAs, all of which were upregulated in carcinoma tissue, were associated with differential expression of 48 miRNAs, 41 of which were upregulated and seven were downregulated in carcinoma tissue. In total, there were 290 unique associations, 102 of which had an identified seed match. Ten of the interactions with an identified seed match had a negative beta coefficient. In nine of these interactions, the miRNA was downregulated while the mRNA was upregulated; in one interaction, between hsa-miR-424-3p and *CDC6*, both molecules were upregulated. The identified seed match in these 10 associations supports the theory that these miRNAs target the mRNAs. In the case of hsa-miR-424-3p and *CDC6*, while both molecules are upregulated in carcinoma tissue as compared to normal mucosa, the miRNA is most likely mitigating the upregulation of the expression of the mRNA by another factor, possibly acting as a buffer to maintain expression homeostasis. In interactions with downregulated miRNA expression, mRNA expression could be increased as a result of reduced miRNA-mediated repression. These interactions comprised six unique mRNAs (*CCNA2*, *CDC6*, *MAD2L1*, *PRKDC, SMC1A*, and *YWHAB*) and six unique miRNAs (hsa-miR-145-5p, hsa-miR-150-5p, hsa-miR-195-5p, hsa-miR-375, hsa-miR-650, and hsa-miR-6515-5p).

The other 92 interactions that had an identified seed match displayed positive beta coefficients, indicating that as the mRNA expression increases, the expression of the miRNA increases as well. This type of relationship suggests that these molecules could interact in feedback loops, in which the mRNA influences transcription of the miRNA and the miRNA in turn post-transcriptionally regulates the mRNA, or feed-forward loops (FFL), in which both molecules regulate a third target in addition to regulating one another [8].

One potential FFL can be seen with *E2F5* (FC 1.52) and miRNAs hsa-miR-17-5p (FC 3.73, beta coefficient 0.30) and hsa-miR-20a-5p (FC 4.02, beta coefficient 0.31). Hsa-miR-17-5p and hsa-miR-20a-5p belong to the cluster miR-17~92. E2F1-3 are cited as transcriptional enhancers of miRNAs in the miR-17~92 cluster [7]; E2F4-5 are traditionally transcriptional repressors [9, 10]. However, as activators have shown repressive activity and repressors have shown activating effects [9], it may be that, as we see positive beta coefficients in these interactions, E2F5 acts as a transcriptional enhancer of these miRNA clusters in colorectal cancer.

Transcription of proteins essential for G_1_-S depends on E2F1-5 proteins and their dimerization partners (pRb, p107, and p130, encoded by *RB1*, *RBL1*, and *RBL2* respectively); genes encoding these proteins are often dysregulated or mutated in cancer [9]. P130 has been shown to bind with repressive E2Fs to inhibit E2F involvement in transcription during G_0_/G_1_, and it has been hypothesized that miR-17 can limit this repression [10]. *RBL2* was marginally downregulated in carcinoma tissue; however *RBL1*, which was significantly upregulated (FC = 2.30), was associated significantly with 11 miRNAs in our data, all with positive beta coefficients, and five of these had identified seed matches (hsa-miR-17-5p, hsa-miR-19b-3p, hsa-miR-20a-5p, hsa-miR-20b-5p, and hsa-miR-93-5p). These five miRNAs were associated with the most mRNAs and were often expressed in tandem. They are part of a larger group of miRNAs that derive from three paralogous clusters: miR-17~92 (which includes hsa-miR-17-5p, hsa-miR-20a-5p, hsa-miR-92a-3p), miR-106a~363 (which includes hsa-miR-19b-3p, hsa-miR-20b-5p), and miR-106b~25 (which includes hsa-miR-106b-5p, hsa-miR-25-3p, hsa-miR-93-5p).

The mir-17~92 cluster is known to inhibit translation of *RBL1* as well as *E2F* genes [7]. Phosphorylation of Rb, p107, and p130 by CDK4/6-cyclin D1 (encoded for by *CCND1*) complex inactivates the Rb proteins and allows E2F proteins to regulate transcription of the genes that encode for proteins necessary for S phase, including cyclin E. Cyclin D1 is present in early G_1_ and its production is induced primarily by mitogenic growth factors [3]; it is responsible for progressing the cell past the restriction point. *CCND1* was upregulated in carcinoma tissue and was associated with 11 miRNAs, nine of which had seed matches, including hsa-miR-106b-5p, hsa-miR-17-5p, hsa-miR-20a-5p, hsa-miR-20b-5p, and hsa-miR-93-5p; these miRNAs have been reported to target *CCND1* and be over expressed in colon cancer [7].

As E2Fs can regulate their own transcription, synchronized transcription of these clusters may serve to dampen E2F activity and prevent uncontrolled growth or unchecked apoptosis [10, 11]. The paralogous miRNAs comprised 59 of the 92 (64%) interactions with positive beta coefficients and seed region matches, indicating that these miRNAs constitute a large portion of those potentially involved in feed-forward loops that regulate the cell cycle in colorectal cancer subjects. It has been suggested that the miRNAs in these clusters may act synergistically, by either targeting the same mRNAs or by targeting multiple nodes in the same pathway [12]; these results support a collaborative effect on mRNA expression in the cell cycle pathway by these miRNAs, especially during the G_1_-S transition.

As shown in Figure 1, E2F5 in conjunction with p107 (encoded by *RBL1*) downregulate *MYC* at the start of the cell cycle in G_1_. MYC, a transcriptional regulator itself, controls the production of many proteins as well as non-coding RNAs, including miRNAs [7], and is at the center of a multitude of FFLs that regulate cell cycle processes [13]. *MYC* was upregulated in carcinoma tissue (FC 3.70) and was associated with both hsa-miR-17-5p and hsa-miR-20a-5p with positive beta coefficients, without identified seed matches. MYC has been reported to directly induce transcription of the miR-17~92 cluster [14]; this is supported by the positive beta coefficients detected between *MYC* and these miRNAs in our data. MYC is also known to directly enhance *E2F* transcription [10]. As repressor E2F proteins can exhibit transcriptional activation, these findings might suggest the presence of an auto-regulatory loop, in which MYC enhances both the miR-17~92 cluster as well as *E2F* transcription, and E2F5 increases transcription of hsa-miR-17-5p and hsa-miR-20a-5p, which in turn post-transcriptionally inhibit *E2F5*, as well as *RBL1*.

Transcription of the mini chromosome matrix (MCM) protein genes, *MCM2-7*, by E2F proteins begins in G_1_ [3]. The proteins comprise the catalytic core of the helicase that unwinds parental DNA to generate the template strands; overexpression of MCM genes has been linked to cancer development [15]. *MCM2-7* were all upregulated in carcinoma tissue in our data, and *MCM3*, *MCM4*, and *MCM6* were associated with differential miRNA expression, including hsa-miR-106b-5p, hsa-miR-17-5p, hsa-miR-19b-3p, hsa-miR-20a-5p, hsa-miR-20b-5p, hsa-miR-25-3p, and hsa-miR-93-5p. *MCM3* and *MCM4* had seed region matches with all of these miRNAs except for hsa-miR-25-3p, and *MCM6* only had a seed match with hsa-miR-25-3p. *MCM4* also had an identified seed match with hsa-miR-130b-3p. The miR-106b~25 cluster resides in the intron of *MCM7* and these are co-transcribed [16]; however, we did not detect these expected expression associations. CDC6 is part of the pre-replicative complex and its presence facilitates MCM protein loading onto chromosomes [3]. It is primarily expressed in the nucleus during G_1_, becoming inactivated by cyclin A-CDK2-mediated phosphorylation and relocated to the cytoplasm at the onset of S phase [3]. Expression of *CDC6* was associated with hsa-miR-145-5p, hsa-miR-195-5p, hsa-miR-196a-5p, hsa-miR-424-3p, and hsa-miR-93-5p. Hsa-miR-145-5p, hsa-miR-195-5p, and hsa-miR-424-3p were associated with negative beta coefficients. These miRNAs were downregulated in carcinoma tissue, with the exception of hsa-miR-424-3p, which was upregulated, and hsa-miR-195-5p, hsa-miR-196a-5p, and hsa-miR-424-3p had identified seed matches with *CDC6*.

Also in late G_1_, cyclin E participates in the phosphorylation of Rb and release of E2F; subsequently in S phase, cyclin E-CDK2 phosphorylates components of the prereplication complex, enabling DNA replication initiation [3]. Although *CCNE1*, *CCNE2*, and *CDK2* were upregulated in carcinoma tissue in our data (FC = 1.81, 1.26, 1.83 respectively), *CCNE1* and *CDK2* were not associated with differential miRNA expression, and *CCNE2* was not evaluated with miRNA expression. As the cell progresses into S phase, DNA replication is initiated and transcriptional regulators are inhibited to turn off gene transcription, which may be facilitated by FFLs involving the paralogous miRNA clusters. *SKP2*, which encodes a protein in the ubiquitin ligase regulatory complex, regulates the stability of E2F proteins in S and G_2_; E2F proteins target *SKP2*, constituting a negative FFL [9]. *SKP2* was associated with hsa-miR-25-3p and hsa-miR-93-5p, which belong to the miR-106b~25 cluster. Cyclin A has been implicated in S phase regulation, as its presence promotes S phase progression, and it is able to bind mitotic spindles independent of CDKs [3, 17]. *CCNA2* was associated with differential expression of nine miRNAs: hsa-miR-106b-5p, hsa-miR-17-5p, hsa-miR-196a-5p, hsa-miR-20b-5p, hsa-miR-25-3p, and hsa-miR-93-5p with positive beta coefficients and no seed matches; hsa-miR-150-5p and hsa-miR-650 with negative beta coefficients, the former with an identified seed match; and hsa-miR-130b-3p with a positive beta coefficient and an identified seed match.

The G_2_-M phase transition is initiated when CDC25C dephosphorylates and activates cyclin B1-CDK1 complexes, which then translocate to the nucleus and initiate mitosis [4]. *CDC25C* was upregulated in carcinoma tissue and associated with the paralogous cluster miRNAs, but no seed matches were identified; it is possible that the same regulator that increases the expression of these miRNAs influences *CDC25C* expression. *CCNB1*, encoding for cyclin B1, was associated with differential expression of four miRNAs: hsa-miR-145-5p and hsa-miR-195-5p were associated with negative beta coefficients, while hsa-miR-25-3p and hsa-miR-93-5p were associated with positive beta coefficients; the latter also had an identified seed match.

The 14-3-3 proteins, encoded for by *YWHAB*, which had seed matches with 19 miRNAs, and *YWHAQ*, which had seed matches with seven miRNAs, bind to and inhibit translation of *CDC25B* and *CDC25C*, which are needed for mitotic entry [18]. The differentially expressed miRNAs with identified seed matches included the paralogous cluster miRNAs, as well as hsa-miR-1246, hsa-miR-130b-3p, hsa-miR-196a-5p, hsa-miR-196b-5p, hsa-miR-199a-3p, hsa-miR-21-3p, hsa-miR-221-3p, hsa-miR-24-3p, hsa-miR-27a-3p, hsa-miR-29b-3p, hsa-miR-32-3p, hsa-miR-361-5p, hsa-miR-425-5p, and hsa-miR-501-3p with positive beta coefficients and hsa-miR-375 and hsa-miR-6515-5p with negative beta coefficients. *YWHAG* was associated with four miRNAs with identified seed matches (hsa-miR-21-3p, hsa-miR-221-3p, hsa-miR-27a-3p, and hsa-miR-29b-3p), and 10 others without seed matches, including many of the paralogous cluster miRNAs. Together, these results suggest that these miRNAs assist in the cell cycle's progression into M-phase.

During metaphase, chromosomes attach to microtubules, *via* kinetochores on the sister chromatids, and become properly oriented; this is known as the Spindle Assembly Checkpoint (SAC) [19]. Unattached kinetochores catalyze the formation of the Mitotic Checkpoint Complex (MCC), consisting of proteins encoded by *BUB1B*, *BUB1*, and *MAD2L1*, which inhibits the CDC20 subunit of the Anaphase Promoting Complex (APC); recruitment of the MCC and activation of the SAC are dependent on BUB1, a mitotic kinase [19]. *BUB1* was associated with differential expression of hsa-miR-93-5p, and a seed match was identified. *BUB1B* was associated with hsa-miR-145-5p with a negative beta coefficient, with no identified seed match. Both *BUB3* and *MAD2L1* were positively associated with hsa-miR-106b-5p, hsa-miR-19b-3p, hsa-miR-20b-5p, hsa-miR-25-3p, and hsa-miR-93-5p, with seed matches identified for all except hsa-miR-25-3p. *MAD2L1* was additionally associated with hsa-miR-130b-3p, hsa-miR-196a-5p, hsa-miR-501-3p (positive beta coefficient, no seed match), hsa-miR-17-5p, hsa-20a-5p and hsa-miR-583 (positive beta coefficient, identified seed match), hsa-miR-650 and hsa-miR-145-5p (negative beta coefficient, identified seed match), and hsa-miR-195-5p (negative beta coefficient, no seed match).

The APC is required for progression through and exit from mitosis. CDC20, initiates anaphase by ubiquitinating securin, thus enabling chromatin separation; after anaphase begins, the CDC20 subunit is degraded by CDH1, encoded by *FZR1* [20]. Ubiquitination and subsequent degradation of cyclin B by APC is a crucial step for mitotic exit [21]. *FZR1* was slightly downregulated in our data, FC = 0.93, and not evaluated with differential miRNA expression. *ANAPC1* and *CDC20* were both upregulated, and associated with differential miRNA expression, as was *CDC16*; none of these genes had identified seed matches. Both *ANAPC1* and *CDC16* were associated with hsa-miR-17-5p, hsa-miR-19b-3p, hsa-miR-20a-5p, hsa-miR-20b-5p, and hsa-miR-92a-3p; *ANAPC1* was also associated with hsa-miR-196a-5p and hsa-miR-93-5p; and *CDC16* was associated with hsa-miR-151a-3p, hsa-miR-15a-5p, hsa-miR-199b-5p, and hsa-miR-361-5p. All of these associations displayed positive beta coefficients. Increased *CDC20* was associated with decreased differential expression of hsa-miR-145-5p.

All eight of the paralogous-cluster miRNAs (hsa-miR-106b-5p, hsa-miR-17-5p, hsa-miR-19b-3p, hsa-miR-20a-5p, hsa-miR-20b-5p, hsa-miR-25-3p, hsa-miR-92a-3p, and hsa-miR-93-5p) were associated with *RAD21*, a DNA repair gene, and all but hsa-miR-19b-3p had an identified seed match. Involved in the M-phase, *RAD21* was overexpressed in carcinoma tissue; decreased levels of RAD21 have been reported to be associated with loss of cell proliferation in breast cancer [22].

Four mRNAs and 12 miRNAs were associated with altered colorectal cancer survival prior to adjustment for multiple comparisons. No mRNAs remained associated after adjusting for multiple comparisons; two miRNAs, hsa-miR-20b-5p (FDR = 0.01) and hsa-miR-15a-5p (FDR = 0.02), remained significant after adjustment, but had no identifying seed match with any mRNAs. These miRNAs may serve as useful biomarkers for prognosis, given their association with improved colorectal cancer survival when differential expression increased.

MiR-15a, which belongs to the mir-15a-16-1 cluster, is reported to have anti-proliferative properties, in that it halts the cell cycle in G_1_ by targeting genes encoding for CDK1, 2 and 6 and cyclins D1, D3 and E1 [7]. In our data, hsa-miR-15a-5p was associated with *CDC16*, *RAD21*, and *YWHAB* with positive beta coefficients and no identified seed matches. MiR-34a has been associated with CDK4, CDK6, cyclins D and E2, E2F1 and E2F3, and MYC in other studies [7]; however we did not detect these associations. Instead, we identified interactions between hsa-miR-34a and *PRKDC, RAD21,* and *YWHAB* with positive beta coefficients and no identified seed matches.

One potential limitation of our study is that we chose to limit our analysis of miRNAs to genes with a FC of > 1.50 or < 0.67, and as such we did not evaluate cell cycle signaling genes whose FC fell outside this range with miRNA expression. Such genes include *E2F2/4*, *CCND3*, *CCNE2*, *CDKN1B*, *SMAD2-4*, and *TGFB1/3*. This was done to reduce statistical noise and condense our analyses to involve genes with potentially a greater biological impact; however, expression of genes with smaller FC may also be influenced by miRNAs. MiRNAs have been described as ‘fine-tuners’ of expression, which work to maintain homeostasis; as such, it could be that small changes in expression would reflect meaningful regulatory interactions [23]. We also replicated significantly differentially expressed miRNAs cited in the literature, such as miR-34a and miR-15a; however, we did not detect the same associations with mRNA expression as in the literature. This may be due to our restrictions on FC, or it may be that these associations are not present in colorectal cancer cases. Our paired dataset of miRNA and mRNA data, along with survival data, enable us to integrate many components of the carcinogenesis process, and to determine which associations most likely have a greater effect on outcomes. Using paired normal colorectal mucosa and carcinoma colorectal tissue allowed us to control for variations in collection, storage, and processing. We were unable to look at protein levels, and as such cannot discern for certain the impact miRNAs have on associated mRNAs; however, by identifying seed matches between miRNA and mRNA 3’ UTR sequences, we are able to better predict immediate *versus* indirect or downstream relationships. We consider our use of a microarray platform and RNA-seq data to be an asset as well. By using such instruments, we were able to take a discovery approach, and investigate large-scale miRNA and mRNA dysregulation. Few mRNAs in our data were associated significantly with altered colorectal cancer survival after adjustment for multiple comparisons, which may be due in part to our smaller sample size. We encourage others to replicate these findings in other data sets.

Our findings suggest that miRNAs may impact mRNA translation at multiple levels within the cell cycle. Transcription of the miRNAs in the paralogous clusters miR-17~92 miR-106a~363, and miR-106b~25, in particular, appear to regulate the G_1_-S phases and G_1_-to-S transition, through E2F and MYC feedback and feed-forward loops. Their direct association with other mRNAs without the presence of an identified seed match in other phases of the cell cycle may indicate joint regulation of these miRNAs and mRNAs. M phase may also be regulated by miRNAs, as numerous components of the SAC and APC are associated with differential miRNA expression. This investigation provides a broad overview of miRNA and mRNA activity involved in cell cycle signaling in colorectal cancer cases. While specific interactions are difficult to decipher, our analyses provide beneficial insight into the interconnectedness of cell cycle regulation, and identifies potential biomarkers for prognosis.

## MATERIALS AND METHODS

### Study participants

Study participants came from two population-based case-control studies that included all incident colon and rectal cancer between 30 to 79 years of age in Utah or were health plan members of Kaiser Permanente Northern California (KPNC). Participants were non-Hispanic white, Hispanic, or black for the colon cancer study; the rectal cancer study also included people of Asian race [24, 25]. Case diagnosis was verified by tumor registry data as a first primary adenocarcinoma of the colon or rectum and occurred between October 1991 and September 1994 (colon study) and between May 1997 and May 2001 (rectal study) [26]. The Institutional Review Boards (IRB) at the University of Utah and at KPNC approved the study.

### Survival data

Survival information was obtained from Surveillance, Epidemiology, and End Results (SEER) tumor registries in Utah and California. Survival months were calculated from the date of diagnosis to the date of last contact or death. AJCC stage and cause of death also were obtained from the SEER registries. We assessed colorectal cancer-specific mortality. Individuals who died from other causes were censored at the time of death. Individuals alive at the end of follow-up were also censored at the time of last follow-up, which was December of 2001 for colon cancer subjects and April of 2007 for rectal cancer subjects, when calculating survival months.

### RNA processing

Formalin-fixed paraffin embedded tissue from the initial biopsy or surgery was used to extract RNA. RNA was then isolated and purified from carcinoma tissue and adjacent normal mucosa as previously described [27]. We observed no differences in RNA quality based on age of the tissue.

#### mRNA: RNA-Seq sequencing library preparation and data processing

Total RNA from 245 colorectal carcinoma and normal mucosa pairs was chosen for sequencing based on availability of RNA and high quality miRNA data; 217 pairs passed quality control (QC) and are used in these analyses. RNA library construction was done with the Illumina TruSeq Stranded Total RNA Sample Preparation Kit with Ribo-Zero. The samples were then fragmented and primed for cDNA synthesis, adapters were then ligated onto the cDNA, and the resulting samples were then amplified using PCR; the amplified library was then purified using Agencount AMPure XP beads. A more detailed description of the methods can be found in our previous work [28]. Illumina TruSeq v3 single read flow cell and a 50 cycle single-read sequence run was performed on an Illumina HiSeq instrument. Reads were aligned to a sequence database containing the human genome (build GRCh37/hg19, February 2009 from genome.ucsc.edu) and alignment was performed using novoalign v2.08.01. Total gene counts were calculated for each exon and UTR of the genes using a list of gene coordinates obtained from http://genome.ucsc.edu. We disregarded genes that were not expressed in our RNA-Seq data or for which the expression was missing for the majority of samples [28].

### miRNA

The Agilent Human miRNA Microarray V19.0 was used. Data were required to pass stringent QC parameters established by Agilent that included tests for excessive background fluorescence, excessive variation among probe sequence replicates on the array, and measures of the total gene signal on the array to assess low signal. Samples that failed to meet quality standards were re-labeled, hybridized to arrays, and re-scanned. If a sample failed QC assessment a second time, the sample was excluded from the analysis. The repeatability associated with this microarray was extremely high (*r* = 0.98) [26]; comparison of miRNA expression levels obtained from the Agilent microarray to those obtained from qPCR had an agreement of 100% in terms of directionality of findings and the FCs were almost identical [29]. To normalize differences in miRNA expression that could be attributed to the array, amount of RNA, location on array, or factors that could erroneously influence miRNA expression levels, total gene signal was normalized by multiplying each sample by a scaling factor which was the median of the 75^th^ percentiles of all the samples divided by the individual 75^th^ percentile of each sample [30].

### Cell cycle signaling genes

The Kyoto Encyclopedia of Genes and Genomes (KEGG) (http://www.kegg.jp/kegg-bin/show_pathway?map04110) pathway map for Cell Cycle signaling was used to identify genes associated with this pathway. Using this map, we identified 124 genes (Supplementary Table 1), of which we were able to analyze 123 that were expressed sufficiently in colorectal tissue.

### Statistical methods

We utilized a negative binomial mixed effects model in SAS (accounting for carcinoma/normal status as well as for subject effect) to determine genes in the cell cycle pathway that had a significant difference in expression between individually paired colorectal carcinoma and normal mucosa and related fold changes (FC). In this test, we offset the overall exposure as the expression of all identified protein-coding genes (*n* = 17461). The Benjamini and Hochberg [31] procedure was used to control the false discovery rate (FDR) using a value of 0.05 or less. An FC greater than one indicates a positive differential expression (i.e. up-regulated in carcinoma), while an FC between zero and one indicates a negative differential expression (i.e. down-regulated in carcinoma). We determined expression level of each gene by dividing the total expression for that gene in an individual by the total expression of all protein-coding genes per million transcripts (RPMPCG or reads per million protein-coding genes). We focused on those genes with an FC of > 1.50 or < 0.67 for analysis with miRNAs, under the assumption that these levels of FC may have a greater biological significance than FCs closer to one. There were 814 miRNAs expressed in greater than 20% of normal colorectal mucosa that were analyzed; differential expression was calculated using subject-level paired data as the expression in the carcinoma tissue minus the expression in the normal mucosa. In these analyses, we fit a least squares linear regression model to the RPMPCG differential expression levels and miRNA differential expression levels. P-values were generated using the bootstrap method by creating a distribution of 10,000 F statistics derived by resampling the residuals from the null hypothesis model of no association between gene expression and miRNA expression using the boot package in R. Linear models were adjusted for age and sex. Multiple comparison adjustments for gene/miRNA associations were made at the gene level using the FDR by Benjamini and Hochberg [31].

We performed survival analysis for all mRNAs that were significantly differentially expressed as well as miRNAs that were associated with differential mRNA expression. The R package “survival” was used to calculate p-values based upon 10,000 permutations of the likelihood ratio test from the Cox proportional hazards model adjusted for age at diagnosis, sex, and AJCC tumor stage. We report hazard ratios (HR), with the unit of change being the interquartile range (IQR) of differential expression, and 95% confidence intervals (CI). The IQR was chosen as the unit of change as it provides a more standard unit of change across various mRNA and miRNA expression levels. Thus, rather than use the unit of expression for each mRNA/miRNA, which can vary greatly in terms of meaning when interpreting the HR, we have used the IQR of expression.

### Bioinformatics analysis

We determined seed region pairings between miRNA and mRNA by analyzing the mRNA 3’ UTR FASTA as well as the seed region sequence of the associated miRNA. As described in our previous work [32], we calculated and included seeds of six, seven, and eight nucleotides in length. A seed match would increase the probability that identified genes associated with specific miRNAs are more likely to have a direct association, given a higher propensity for binding and thus mRNA degradation. As miRTarBase [33] uses findings from many different investigations spanning across years and alignments, we used FASTA sequences generated from both GRCh37 and GRCh38 Homo sapiens alignments, using UCSC Table Browser (https://genome.ucsc.edu/cgi-bin/hgTables) [34]. We downloaded FASTA sequences that matched our Ensembl IDs and had a consensus coding sequences (CCDS) available. Analysis was conducted using scripts in R 3.2.3 and in perl 5.018002.

## SUPPLEMENTARY MATERIALS TABLE


