# Supplementary material for: miRNA involvement in cell cycle regulation in colorectal cancer cases
Source: Genes Cancer. 2018 Jan;9(1-2):53–65. doi: 10.18632/genesandcancer.167 (PMC5931252; doi:10.18632/genesandcancer.167)
Supplement: Supplementary file 1 [file ganc-09-053-s001.pdf]

MiRNA involvement in cell cycle regulation in colorectal cancer cases – Mullany et al

Supplementary Table 1: MiRNA-mRNA associations in colorectal cancer.

	Mean Expression				Mean Expression				P-value	
mRNA	Carcinoma	Normal Mucosa	Fold Change	miRNA	Carcinoma	Normal Mucosa	Fold Change	Beta	Raw	FDR
ANAPC1	75.40	43.01	1.75	hsa-miR-17-5p	61.04	16.38	3.73	0.29	0.0003	0.0349
				hsa-miR-196a-5p	6.70	4.21	1.59	0.25	0.0003	0.0349
				hsa-miR-19b-3p	29.80	10.42	2.86	0.26	0.0003	0.0349
				hsa-miR-20a-5p	70.78	17.61	4.02	0.27	<.0001	0.0349
				hsa-miR-20b-5p	17.65	3.30	5.35	0.26	0.0002	0.0349
				hsa-miR-92a-3p	121.60	41.18	2.95	0.27	0.0003	0.0349
				hsa-miR-93-5p	41.72	15.20	2.74	0.25	0.0003	0.0349
BUB1	53.35	18.38	2.90	**hsa-miR-93-5p**	41.72	15.20	2.74	0.26	0.0002	0.0488
BUB1B	41.59	17.15	2.42	hsa-miR-145-5p	132.97	223.14	0.60	-0.27	0.0002	0.0407
BUB3	94.35	60.85	1.55	**hsa-miR-106b-5p**	15.90	5.19	3.06	0.30	<.0001	0.0136
				**hsa-miR-19b-3p**	29.80	10.42	2.86	0.26	0.0003	0.0244
				**hsa-miR-20b-5p**	17.65	3.30	5.35	0.25	<.0001	0.0136
				hsa-miR-25-3p	30.05	12.78	2.35	0.35	<.0001	0.0136
				**hsa-miR-93-5p**	41.72	15.20	2.74	0.30	<.0001	0.0136
CCNA2	40.09	14.62	2.74	hsa-miR-106b-5p	15.90	5.19	3.06	0.25	0.0002	0.0163
				**hsa-miR-130b-3p**	8.74	4.89	1.79	0.28	0.0003	0.0222
				**hsa-miR-150-5p**	14.90	39.17	0.38	-0.28	<.0001	0.0163
				hsa-miR-17-5p	61.04	16.38	3.73	0.27	0.0005	0.0313
				hsa-miR-196a-5p	6.70	4.21	1.59	0.25	0.0002	0.0163
				hsa-miR-20b-5p	17.65	3.30	5.35	0.30	<.0001	0.0163
				hsa-miR-25-3p	30.05	12.78	2.35	0.27	0.0002	0.0163
				hsa-miR-650	4.51	16.60	0.27	-0.24	0.0007	0.0407
				hsa-miR-93-5p	41.72	15.20	2.74	0.33	<.0001	0.0163
CCNB1	32.40	9.25	3.50	hsa-miR-145-5p	132.97	223.14	0.60	-0.30	<.0001	0.0203
				hsa-miR-195-5p	3.59	12.18	0.29	-0.28	0.0003	0.0305
				hsa-miR-25-3p	30.05	12.78	2.35	0.27	0.0002	0.0233
				**hsa-miR-93-5p**	41.72	15.20	2.74	0.30	<.0001	0.0203
CCND1	317.79	122.64	2.59	**hsa-miR-106b-5p**	15.90	5.19	3.06	0.25	0.0006	0.0349
				**hsa-miR-17-5p**	61.04	16.38	3.73	0.30	<.0001	0.0244
				**hsa-miR-19b-3p**	29.80	10.42	2.86	0.28	0.0003	0.0244
				hsa-miR-203a	12.52	3.70	3.38	0.27	0.0002	0.0244
				**hsa-miR-20a-5p**	70.78	17.61	4.02	0.28	<.0001	0.0244
				**hsa-miR-20b-5p**	17.65	3.30	5.35	0.29	<.0001	0.0244
				hsa-miR-21-5p	463.11	167.37	2.77	0.25	0.0006	0.0349
				**hsa-miR-221-3p**	13.53	4.12	3.28	0.26	0.0003	0.0244
				**hsa-miR-27a-3p**	56.26	23.29	2.42	0.27	0.0002	0.0244
				**hsa-miR-29b-3p**	24.31	9.83	2.47	0.27	0.0003	0.0244
				**hsa-miR-93-5p**	41.72	15.20	2.74	0.26	0.0002	0.0244
CDC16	90.37	58.88	1.53	hsa-miR-151a-3p	5.15	1.56	3.31	0.23	0.0011	0.0422
				hsa-miR-15a-5p	7.69	5.07	1.52	0.29	<.0001	0.0407
				hsa-miR-17-5p	61.04	16.38	3.73	0.24	0.001	0.0422
				hsa-miR-199b-5p	4.69	1.53	3.07	0.23	0.0009	0.0422
				hsa-miR-19b-3p	29.80	10.42	2.86	0.23	0.0004	0.0422
				hsa-miR-20a-5p	70.78	17.61	4.02	0.26	0.0003	0.0407
				hsa-miR-20b-5p	17.65	3.30	5.35	0.27	0.0003	0.0407

				hsa-miR-361-5p	11.62	6.20	1.87	0.24	0.0011	0.0422
				hsa-miR-92a-3p	121.60	41.18	2.95	0.36	<.0001	0.0407
CDC20	22.28	10.17	2.19	hsa-miR-145-5p	132.97	223.14	0.60	-0.29	<.0001	0.0271
CDC25A	21.63	10.61	2.04	**hsa-miR-196a-5p**	6.70	4.21	1.59	0.28	<.0001	0.0271
CDC25C	7.95	3.46	2.30	hsa-miR-106b-5p	15.90	5.19	3.06	0.29	0.0002	0.0407
				hsa-miR-145-5p	132.97	223.14	0.60	-0.24	0.0004	0.0452
				hsa-miR-17-5p	61.04	16.38	3.73	0.29	0.0005	0.0452
				hsa-miR-19b-3p	29.80	10.42	2.86	0.24	0.0006	0.0488
				hsa-miR-203a	12.52	3.70	3.38	0.28	<.0001	0.0407
				hsa-miR-20b-5p	17.65	3.30	5.35	0.28	0.0003	0.0452
				hsa-miR-25-3p	30.05	12.78	2.35	0.27	0.0002	0.0407
				hsa-miR-501-3p	7.07	2.95	2.39	0.25	0.0005	0.0452
				hsa-miR-93-5p	41.72	15.20	2.74	0.32	<.0001	0.0407
CDC6	32.30	11.33	2.85	hsa-miR-145-5p	132.97	223.14	0.60	-0.33	<.0001	0.0203
				**hsa-miR-195-5p**	3.59	12.18	0.29	-0.26	0.0003	0.0271
				**hsa-miR-196a-5p**	6.70	4.21	1.59	0.26	0.0002	0.0203
				**hsa-miR-424-3p**	39.81	25.37	1.57	-0.26	0.0006	0.0444
				hsa-miR-93-5p	41.72	15.20	2.74	0.25	0.0006	0.0444
CDK1	41.36	11.94	3.46	hsa-miR-106b-5p	15.90	5.19	3.06	0.24	0.0006	0.0488
				hsa-miR-17-5p	61.04	16.38	3.73	0.28	<.0001	0.0203
				hsa-miR-19b-3p	29.80	10.42	2.86	0.29	<.0001	0.0203
				hsa-miR-20b-5p	17.65	3.30	5.35	0.27	0.0002	0.0271
				hsa-miR-221-3p	13.53	4.12	3.28	0.24	0.0006	0.0488
				hsa-miR-25-3p	30.05	12.78	2.35	0.35	<.0001	0.0203
				hsa-miR-93-5p	41.72	15.20	2.74	0.33	<.0001	0.0203
CDK4	66.65	26.90	2.48	hsa-miR-17-5p	61.04	16.38	3.73	0.33	<.0001	0.0271
				hsa-miR-20a-5p	70.78	17.61	4.02	0.28	<.0001	0.0271
				hsa-miR-20b-5p	17.65	3.30	5.35	0.26	0.0002	0.0407
				**hsa-miR-3651**	58.66	25.92	2.26	0.26	0.0003	0.0488
				hsa-miR-93-5p	41.72	15.20	2.74	0.33	<.0001	0.0271
E2F5	46.80	30.87	1.52	**hsa-miR-17-5p**	61.04	16.38	3.73	0.30	<.0001	0.0407
				**hsa-miR-20a-5p**	70.78	17.61	4.02	0.31	<.0001	0.0407
ESPL1	39.52	18.82	2.10	hsa-miR-1246	629.21	412.81	1.52	0.30	<.0001	0.0163
				hsa-miR-130b-3p	8.74	4.89	1.79	0.25	0.0004	0.0407
				hsa-miR-17-5p	61.04	16.38	3.73	0.28	0.0002	0.0233
				hsa-miR-19b-3p	29.80	10.42	2.86	0.26	0.0002	0.0233
				hsa-miR-20a-5p	70.78	17.61	4.02	0.26	<.0001	0.0163
				hsa-miR-25-3p	30.05	12.78	2.35	0.33	<.0001	0.0163
				hsa-miR-93-5p	41.72	15.20	2.74	0.31	<.0001	0.0163
MAD2L1	15.96	4.92	3.24	**hsa-miR-106b-5p**	15.90	5.19	3.06	0.29	<.0001	0.0116
				hsa-miR-130b-3p	8.74	4.89	1.79	0.36	<.0001	0.0116
				**hsa-miR-145-5p**	132.97	223.14	0.60	-0.24	0.0008	0.0362
				**hsa-miR-17-5p**	61.04	16.38	3.73	0.28	<.0001	0.0116
				hsa-miR-195-5p	3.59	12.18	0.29	-0.23	0.0008	0.0362
				hsa-miR-196a-5p	6.70	4.21	1.59	0.25	0.0002	0.0136
				**hsa-miR-19b-3p**	29.80	10.42	2.86	0.25	0.0002	0.0136
				**hsa-miR-20a-5p**	70.78	17.61	4.02	0.25	0.0005	0.0271
				**hsa-miR-20b-5p**	17.65	3.30	5.35	0.33	<.0001	0.0116
				hsa-miR-25-3p	30.05	12.78	2.35	0.29	<.0001	0.0116
				hsa-miR-501-3p	7.07	2.95	2.39	0.27	<.0001	0.0116
				**hsa-miR-583**	6.61	3.22	2.05	0.23	0.0009	0.0386
				**hsa-miR-650**	4.51	16.60	0.27	-0.28	<.0001	0.0116
				**hsa-miR-93-5p**	41.72	15.20	2.74	0.31	0.0002	0.0136
MCM3	107.53	41.43	2.60	**hsa-miR-106b-5p**	15.90	5.19	3.06	0.28	<.0001	0.0136
				**hsa-miR-17-5p**	61.04	16.38	3.73	0.34	<.0001	0.0136
				hsa-miR-19b-3p	29.80	10.42	2.86	0.30	<.0001	0.0136

				**hsa-miR-20a-5p**	70.78	17.61	4.02	0.31	<.0001	0.0136
				**hsa-miR-20b-5p**	17.65	3.30	5.35	0.27	0.0003	0.0271
				hsa-miR-221-3p	13.53	4.12	3.28	0.26	0.0002	0.0233
				hsa-miR-25-3p	30.05	12.78	2.35	0.33	<.0001	0.0136
				hsa-miR-29b-3p	24.31	9.83	2.47	0.26	0.0003	0.0271
				**hsa-miR-93-5p**	41.72	15.20	2.74	0.38	<.0001	0.0136
MCM4	115.85	43.65	2.65	**hsa-miR-106b-5p**	15.90	5.19	3.06	0.32	<.0001	0.0102
				**hsa-miR-130b-3p**	8.74	4.89	1.79	0.25	0.0005	0.0313
				hsa-miR-145-5p	132.97	223.14	0.60	-0.26	<.0001	0.0102
				**hsa-miR-17-5p**	61.04	16.38	3.73	0.35	<.0001	0.0102
				hsa-miR-19b-3p	29.80	10.42	2.86	0.33	<.0001	0.0102
				**hsa-miR-20a-5p**	70.78	17.61	4.02	0.31	<.0001	0.0102
				**hsa-miR-20b-5p**	17.65	3.30	5.35	0.30	<.0001	0.0102
				hsa-miR-221-3p	13.53	4.12	3.28	0.27	0.0002	0.0148
				hsa-miR-25-3p	30.05	12.78	2.35	0.31	<.0001	0.0102
				hsa-miR-29b-3p	24.31	9.83	2.47	0.27	0.0002	0.0148
				hsa-miR-650	4.51	16.60	0.27	-0.26	0.0006	0.0349
				**hsa-miR-93-5p**	41.72	15.20	2.74	0.36	<.0001	0.0102
MCM6	55.02	23.15	2.38	hsa-miR-106b-5p	15.90	5.19	3.06	0.32	<.0001	0.0102
				hsa-miR-1246	629.21	412.81	1.52	0.27	<.0001	0.0102
				hsa-miR-17-5p	61.04	16.38	3.73	0.33	<.0001	0.0102
				hsa-miR-19b-3p	29.80	10.42	2.86	0.27	0.0002	0.0181
				hsa-miR-20a-5p	70.78	17.61	4.02	0.28	<.0001	0.0102
				hsa-miR-20b-5p	17.65	3.30	5.35	0.29	<.0001	0.0102
				hsa-miR-221-3p	13.53	4.12	3.28	0.29	<.0001	0.0102
				**hsa-miR-25-3p**	30.05	12.78	2.35	0.36	<.0001	0.0102
				hsa-miR-93-5p	41.72	15.20	2.74	0.40	<.0001	0.0102
MYC	181.11	49.00	3.70	hsa-miR-1246	629.21	412.81	1.52	0.27	0.0002	0.0163
				hsa-miR-17-5p	61.04	16.38	3.73	0.35	<.0001	0.0136
				hsa-miR-19b-3p	29.80	10.42	2.86	0.27	0.0002	0.0163
				hsa-miR-20a-5p	70.78	17.61	4.02	0.33	<.0001	0.0136
				hsa-miR-20b-5p	17.65	3.30	5.35	0.31	0.0002	0.0163
				hsa-miR-3651	58.66	25.92	2.26	0.28	0.0003	0.0188
				hsa-miR-375	20.50	54.53	0.38	-0.29	<.0001	0.0136
				hsa-miR-501-3p	7.07	2.95	2.39	0.26	0.0003	0.0188
				hsa-miR-583	6.61	3.22	2.05	0.26	0.0004	0.0233
				hsa-miR-663a	374.83	234.91	1.60	0.28	0.0003	0.0188
				hsa-miR-663b	65.50	32.21	2.03	0.33	<.0001	0.0136
				hsa-miR-92a-3p	121.60	41.18	2.95	0.32	<.0001	0.0136
PRKDC	927.46	395.35	2.35	**hsa-miR-106b-5p**	15.90	5.19	3.06	0.35	<.0001	0.0037
				hsa-miR-1246	629.21	412.81	1.52	0.35	<.0001	0.0037
				hsa-miR-130b-3p	8.74	4.89	1.79	0.32	<.0001	0.0037
				**hsa-miR-150-5p**	14.90	39.17	0.38	-0.25	0.0005	0.0116
				hsa-miR-151a-3p	5.15	1.56	3.31	0.29	<.0001	0.0037
				**hsa-miR-17-5p**	61.04	16.38	3.73	0.46	<.0001	0.0037
				hsa-miR-196a-5p	6.70	4.21	1.59	0.26	<.0001	0.0037
				hsa-miR-19b-3p	29.80	10.42	2.86	0.40	<.0001	0.0037
				**hsa-miR-203a**	12.52	3.70	3.38	0.26	0.0003	0.0076
				**hsa-miR-20a-5p**	70.78	17.61	4.02	0.43	<.0001	0.0037
				**hsa-miR-20b-5p**	17.65	3.30	5.35	0.41	<.0001	0.0037
				hsa-miR-21-3p	22.68	9.89	2.29	0.23	0.0007	0.0146
				hsa-miR-21-5p	463.11	167.37	2.77	0.27	0.0002	0.006
				hsa-miR-221-3p	13.53	4.12	3.28	0.34	<.0001	0.0037
				**hsa-miR-23a-3p**	174.68	87.53	2.00	0.24	0.0011	0.0218
				hsa-miR-24-3p	106.75	62.39	1.71	0.24	0.0013	0.0225
				hsa-miR-25-3p	30.05	12.78	2.35	0.36	<.0001	0.0037

				**hsa-miR-27a-3p**	56.26	23.29	2.42	0.26	<.0001	0.0037
				hsa-miR-29a-3p	110.29	51.04	2.16	0.27	0.0002	0.006
				hsa-miR-29b-3p	24.31	9.83	2.47	0.33	<.0001	0.0037
				hsa-miR-324-5p	5.20	2.27	2.29	0.21	0.0024	0.0369
				hsa-miR-34a-5p	25.15	12.32	2.04	0.24	0.0005	0.0116
				hsa-miR-361-5p	11.62	6.20	1.87	0.25	0.0003	0.0076
				hsa-miR-3651	58.66	25.92	2.26	0.33	<.0001	0.0037
				hsa-miR-365a-3p	8.43	4.33	1.94	0.20	0.0035	0.0467
				hsa-miR-425-5p	11.76	6.97	1.69	0.28	<.0001	0.0037
				hsa-miR-429	13.33	8.29	1.61	0.25	0.0003	0.0076
				hsa-miR-501-3p	7.07	2.95	2.39	0.29	<.0001	0.0037
				hsa-miR-650	4.51	16.60	0.27	-0.30	<.0001	0.0037
				hsa-miR-663b	65.50	32.21	2.03	0.31	<.0001	0.0037
				hsa-miR-92a-3p	121.60	41.18	2.95	0.38	<.0001	0.0037
				**hsa-miR-93-5p**	41.72	15.20	2.74	0.40	<.0001	0.0037
RAD21	257.68	139.20	1.85	**hsa-miR-106b-5p**	15.90	5.19	3.06	0.37	<.0001	0.0043
				hsa-miR-1246	629.21	412.81	1.52	0.22	0.0021	0.0427
				hsa-miR-151a-3p	5.15	1.56	3.31	0.27	0.0003	0.0094
				hsa-miR-15a-5p	7.69	5.07	1.52	0.23	0.0013	0.0302
				**hsa-miR-17-5p**	61.04	16.38	3.73	0.39	<.0001	0.0043
				hsa-miR-196a-5p	6.70	4.21	1.59	0.25	0.0009	0.0229
				hsa-miR-19b-3p	29.80	10.42	2.86	0.42	<.0001	0.0043
				**hsa-miR-203a**	12.52	3.70	3.38	0.35	<.0001	0.0043
				**hsa-miR-20a-5p**	70.78	17.61	4.02	0.38	<.0001	0.0043
				**hsa-miR-20b-5p**	17.65	3.30	5.35	0.38	<.0001	0.0043
				hsa-miR-21-3p	22.68	9.89	2.29	0.24	0.0006	0.0163
				**hsa-miR-21-5p**	463.11	167.37	2.77	0.27	0.0002	0.0068
				hsa-miR-221-3p	13.53	4.12	3.28	0.31	<.0001	0.0043
				**hsa-miR-25-3p**	30.05	12.78	2.35	0.38	<.0001	0.0043
				hsa-miR-27a-3p	56.26	23.29	2.42	0.24	0.0004	0.0112
				hsa-miR-29a-3p	110.29	51.04	2.16	0.30	<.0001	0.0043
				hsa-miR-29b-3p	24.31	9.83	2.47	0.37	<.0001	0.0043
				hsa-miR-34a-5p	25.15	12.32	2.04	0.26	0.0002	0.0068
				**hsa-miR-3651**	58.66	25.92	2.26	0.32	<.0001	0.0043
				hsa-miR-425-5p	11.76	6.97	1.69	0.31	<.0001	0.0043
				hsa-miR-429	13.33	8.29	1.61	0.29	<.0001	0.0043
				hsa-miR-650	4.51	16.60	0.27	-0.27	0.0002	0.0068
				**hsa-miR-92a-3p**	121.60	41.18	2.95	0.35	<.0001	0.0043
				**hsa-miR-93-5p**	41.72	15.20	2.74	0.36	<.0001	0.0043
RBL1	53.52	23.32	2.30	hsa-miR-1246	629.21	412.81	1.52	0.30	<.0001	0.0116
				**hsa-miR-17-5p**	61.04	16.38	3.73	0.32	<.0001	0.0116
				hsa-miR-196b-5p	17.89	5.53	3.24	0.23	0.0009	0.0407
				**hsa-miR-19b-3p**	29.80	10.42	2.86	0.23	0.0009	0.0407
				**hsa-miR-20a-5p**	70.78	17.61	4.02	0.32	<.0001	0.0116
				**hsa-miR-20b-5p**	17.65	3.30	5.35	0.27	0.0004	0.025
				hsa-miR-25-3p	30.05	12.78	2.35	0.28	0.0002	0.0181
				hsa-miR-3651	58.66	25.92	2.26	0.28	<.0001	0.0116
				hsa-miR-663b	65.50	32.21	2.03	0.25	0.0003	0.0222
				hsa-miR-92a-3p	121.60	41.18	2.95	0.38	<.0001	0.0116
				**hsa-miR-93-5p**	41.72	15.20	2.74	0.28	<.0001	0.0116
SKP2	49.31	24.13	2.04	hsa-miR-25-3p	30.05	12.78	2.35	0.28	0.0002	0.0326
				hsa-miR-93-5p	41.72	15.20	2.74	0.32	<.0001	0.0203
SMC1A	208.24	133.73	1.56	**hsa-miR-145-5p**	132.97	223.14	0.60	-0.26	<.0001	0.0136
				hsa-miR-150-5p	14.90	39.17	0.38	-0.25	0.0002	0.0163
				**hsa-miR-195-5p**	3.59	12.18	0.29	-0.31	<.0001	0.0136
				**hsa-miR-583**	6.61	3.22	2.05	0.24	0.0002	0.0163

TFDP1	119.20	54.04	2.21	hsa-miR-145-5p	132.97	223.14	0.60	-0.25	0.0006	0.0488
				hsa-miR-17-5p	61.04	16.38	3.73	0.33	<.0001	0.0163
				hsa-miR-19b-3p	29.80	10.42	2.86	0.29	0.0002	0.0233
				hsa-miR-20a-5p	70.78	17.61	4.02	0.32	<.0001	0.0163
				hsa-miR-20b-5p	17.65	3.30	5.35	0.31	<.0001	0.0163
				hsa-miR-21-3p	22.68	9.89	2.29	0.29	0.0002	0.0233
				hsa-miR-92a-3p	121.60	41.18	2.95	0.34	<.0001	0.0163
YWHAB	389.26	223.04	1.75	hsa-let-7i-5p	62.16	39.97	1.56	0.22	0.0022	0.0176
				**hsa-miR-106b-5p**	15.90	5.19	3.06	0.30	<.0001	0.0029
				**hsa-miR-1246**	629.21	412.81	1.52	0.24	0.0008	0.0099
				**hsa-miR-130b-3p**	8.74	4.89	1.79	0.18	0.012	0.0485
				hsa-miR-151a-3p	5.15	1.56	3.31	0.30	<.0001	0.0029
				hsa-miR-15a-5p	7.69	5.07	1.52	0.32	0.0002	0.0048
				**hsa-miR-17-5p**	61.04	16.38	3.73	0.44	<.0001	0.0029
				hsa-miR-193b-3p	9.12	5.42	1.68	0.21	0.0036	0.0234
				**hsa-miR-196a-5p**	6.70	4.21	1.59	0.23	0.0007	0.0095
				**hsa-miR-196b-5p**	17.89	5.53	3.24	0.39	<.0001	0.0029
				**hsa-miR-199a-3p**	44.83	22.53	1.99	0.22	0.001	0.0113
				hsa-miR-199a-5p	20.18	9.28	2.17	0.19	0.0057	0.0311
				hsa-miR-199b-5p	4.69	1.53	3.07	0.21	0.0023	0.0177
				**hsa-miR-19b-3p**	29.80	10.42	2.86	0.37	<.0001	0.0029
				**hsa-miR-20a-5p**	70.78	17.61	4.02	0.45	<.0001	0.0029
				**hsa-miR-20b-5p**	17.65	3.30	5.35	0.42	<.0001	0.0029
				**hsa-miR-21-3p**	22.68	9.89	2.29	0.33	<.0001	0.0029
				hsa-miR-21-5p	463.11	167.37	2.77	0.28	0.0002	0.0048
				hsa-miR-221-3p	13.53	4.12	3.28	0.31	<.0001	0.0029
				hsa-miR-23a-3p	174.68	87.53	2.00	0.25	0.0005	0.0083
				**hsa-miR-24-3p**	106.75	62.39	1.71	0.20	0.0067	0.035
				hsa-miR-25-3p	30.05	12.78	2.35	0.33	<.0001	0.0029
				**hsa-miR-27a-3p**	56.26	23.29	2.42	0.28	<.0001	0.0029
				hsa-miR-29a-3p	110.29	51.04	2.16	0.35	<.0001	0.0029
				hsa-miR-29b-3p	24.31	9.83	2.47	0.29	<.0001	0.0029
				**hsa-miR-32-3p**	4.74	2.81	1.68	0.18	0.0115	0.0468
				hsa-miR-34a-5p	25.15	12.32	2.04	0.25	0.0005	0.0083
				**hsa-miR-361-5p**	11.62	6.20	1.87	0.26	0.001	0.0113
				hsa-miR-3651	58.66	25.92	2.26	0.29	<.0001	0.0029
				**hsa-miR-375**	20.50	54.53	0.38	-0.26	0.0002	0.0048
				hsa-miR-424-3p	39.81	25.37	1.57	0.29	<.0001	0.0029
				hsa-miR-425-5p	11.76	6.97	1.69	0.23	0.0007	0.0095
				hsa-miR-4749-3p	8.01	12.04	0.67	-0.21	0.0027	0.0191
				**hsa-miR-501-3p**	7.07	2.95	2.39	0.19	0.0078	0.0376
				**hsa-miR-6515-5p**	1.20	4.41	0.27	-0.25	0.0005	0.0083
				hsa-miR-663a	374.83	234.91	1.60	0.19	0.0056	0.0311
				hsa-miR-663b	65.50	32.21	2.03	0.31	<.0001	0.0029
				hsa-miR-92a-3p	121.60	41.18	2.95	0.51	<.0001	0.0029
				**hsa-miR-93-5p**	41.72	15.20	2.74	0.30	<.0001	0.0029
YWHAE	214.66	141.14	1.52	hsa-miR-424-3p	39.81	25.37	1.57	-0.30	0.0002	0.0407
YWHAG	241.75	119.30	2.03	hsa-miR-106b-5p	15.90	5.19	3.06	0.27	<.0001	0.0116
				hsa-miR-17-5p	61.04	16.38	3.73	0.27	<.0001	0.0116
				hsa-miR-19b-3p	29.80	10.42	2.86	0.26	0.0004	0.0362
				hsa-miR-20a-5p	70.78	17.61	4.02	0.27	0.0002	0.0203
				hsa-miR-20b-5p	17.65	3.30	5.35	0.24	0.0008	0.0465
				**hsa-miR-21-3p**	22.68	9.89	2.29	0.27	<.0001	0.0116
				hsa-miR-21-5p	463.11	167.37	2.77	0.26	0.0006	0.0438
				**hsa-miR-221-3p**	13.53	4.12	3.28	0.29	<.0001	0.0116
				hsa-miR-25-3p	30.05	12.78	2.35	0.28	<.0001	0.0116

				**hsa-miR-27a-3p**	56.26	23.29	2.42	0.26	<.0001	0.0116
				**hsa-miR-29b-3p**	24.31	9.83	2.47	0.24	0.0007	0.0438
				hsa-miR-3651	58.66	25.92	2.26	0.25	0.0006	0.0438
				hsa-miR-650	4.51	16.60	0.27	-0.24	0.0007	0.0438
				hsa-miR-93-5p	41.72	15.20	2.74	0.30	<.0001	0.0116
YWHAH	102.59	66.00	1.55	hsa-miR-650	4.51	16.60	0.27	-0.29	<.0001	0.0163
YWHAQ	130.47	72.74	1.79	**hsa-miR-106b-5p**	15.90	5.19	3.06	0.29	<.0001	0.0116
				**hsa-miR-17-5p**	61.04	16.38	3.73	0.30	<.0001	0.0116
				hsa-miR-19b-3p	29.80	10.42	2.86	0.27	0.0002	0.0181
				**hsa-miR-20a-5p**	70.78	17.61	4.02	0.30	<.0001	0.0116
				**hsa-miR-20b-5p**	17.65	3.30	5.35	0.31	<.0001	0.0116
				hsa-miR-21-3p	22.68	9.89	2.29	0.23	0.0009	0.0471
				hsa-miR-221-3p	13.53	4.12	3.28	0.25	0.0002	0.0181
				hsa-miR-25-3p	30.05	12.78	2.35	0.29	<.0001	0.0116
				**hsa-miR-27a-3p**	56.26	23.29	2.42	0.24	0.001	0.0471
				hsa-miR-29a-3p	110.29	51.04	2.16	0.23	0.0011	0.0471
				hsa-miR-29b-3p	24.31	9.83	2.47	0.24	0.001	0.0471
				hsa-miR-3651	58.66	25.92	2.26	0.24	0.0006	0.0444
				**hsa-miR-425-5p**	11.76	6.97	1.69	0.23	0.0011	0.0471
				hsa-miR-92a-3p	121.60	41.18	2.95	0.23	0.0011	0.0471
				**hsa-miR-93-5p**	41.72	15.20	2.74	0.33	<.0001	0.0116

Bolded items indicate identified seed matches between miRNA and mRNA.
